# Natural product Eriocalyxin B exerts anti-tumor effects by downregulating TCEA3 expression and sensitizes immune checkpoint blockade therapy in osteosarcoma

**DOI:** 10.1590/1414-431X2024e14112

**Published:** 2025-01-31

**Authors:** Ling-Qi Zeng, Mu-Lan Chen, Bin-Bo Fang, Jun-Ze Chen

**Affiliations:** 1 Baiyun Branch, Nanfang Hospital, Southern Medical University, Guangzhou, China; 2 Jingxi Street Community Health Service Center, Baiyun District, Guangzhou, China; 3 Department of Medicine, Taizhou University, Zhejiang, China

**Keywords:** Natural product, Eriocalyxin B, TCEA3, Osteosarcoma

## Abstract

Osteosarcoma (OS) remains the most common bone tumor and the prognosis for many patients remains stagnant due to the unsatisfactory therapeutic effect of conventional treatment regimens. This research explored the effect and mechanism of a novel natural product, Eriocalyxin B (EB), in pathogenesis and immunotherapy in OS. Cell Count Kit 8 assay, colony formation assay, and wound healing assay were employed to detect the proliferative, colony-forming, and migratory abilities of human OS cells following EB treatment. Moreover, xenograft growth assay was performed to assess the effect of EB on OS *in vivo*. Subcutaneous OS models constructed in immunocompetent mice were employed to evaluate the effect of EB treatment in combination with immune checkpoint blockades (ICBs) PD1ab and CTLA4ab. Immunohistochemistry (IHC) staining was utilized to detect the level of CD8^+^ T cells infiltration and Ki67 expression. TARGET database, RNA interference technology, and qPCR assay were employed to explore the mechanism of EB on OS. EB inhibited the proliferative, colony-forming, and migratory abilities of the human OS cells MG63 and U2OS both *in vitro* and *in vivo*. TARGET data analysis demonstrated that up-regulation of TCEA3 was significantly negatively correlated with overall survival in OS patients. EB exerted anti-tumor activity via downregulation of TCEA3. EB, in conjunction with ICBs, synergistically optimized anti-tumorigenic activity against OS in immunocompetent mice. EB may promote infiltration of CD8^+^ T cells and down-regulate Ki67 expression. These results signaled that EB may have a role as a candidate therapeutic or preventive agent for the treatment of OS.

## Introduction

As is well documented, osteosarcoma (OS) remains the most frequent primary bone malignancy and typically afflicts children, adolescents, and young adults, with a median age of 16 years at diagnosis (1-3). Curative surgery remains the mainstay of OS treatment, and commonly used chemotherapeutic agents, including high-dose cisplatin, doxorubicin, ifosfamide, and methotrexate, are recommended to be added to the standard regimen (4). However, due to the high propensity for recurrence and metastasis, the therapeutic effect of current treatment regimens remains unsatisfactory. Indeed, the 5-year overall survival rate for non-metastatic OS patients ranges between 60 to 70%, while that of lung-metastasis patients is merely 20% (5). The lack of response to the standard chemotherapy regimens may be responsible for cancer progression. Increasing the dose of first-line therapy or second-line chemotherapy in patients with a poor response to the chemotherapy has very limited efficacy (6,7). The prognosis for OS patients has stagnated over the last few decades (8). Therefore, there is an urgent need to develop innovative and selective strategies to improve the survival of OS patients.

In recent years, numerous clinical trials have established that immunotherapy is an effective approach for the treatment of human malignancies. Immune checkpoint blockades (ICBs), encompassing monoclonal antibodies, programmed death receptor-1 antibody (PD1ab), and anti-cytotoxic T lymphocyte antigen-4 antibody (CTLA4ab), have been licensed for the treatment of solid tumors, such as bladder cancer, lung cancer, and melanoma, owing to their prolonged, excellent therapeutic effects and mild adverse effects (9). For example, due to the recognizable advantage of ICBs, the Food and Drug Administration (USA) has already approved some ICBs to treat triple-negative breast cancer (TNBC), and developing novel strategy in combination with adenosine A2A receptor antagonists to treat lung cancer, prostate cancer, renal cancer, and related trials are ongoing (9). However, due to the low T cell infiltration in the tumor microenvironment of OS, the majority of OS patients were refractory to ICBs, yielding unsatisfactory outcomes (10).

**Figure 1:**
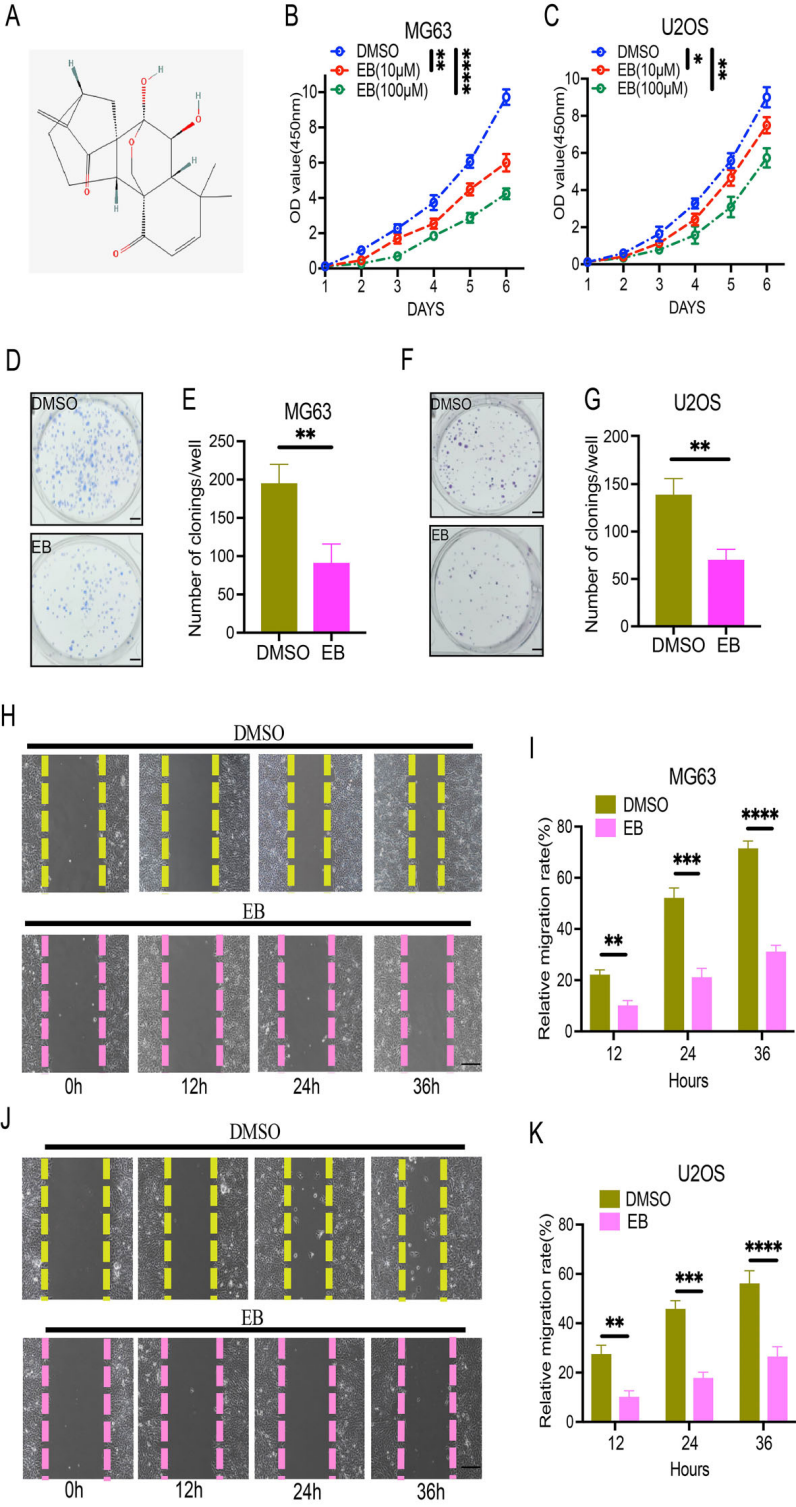
Eriocalyxin B (EB) inhibited the cell proliferative, colony-forming, and migratory abilities of osteosarcoma (OS) cells. **A**, The molecular formula of EB. **B** and **C**, Cell proliferation ability of MG63 (**B**) and U2OS cells (**C**) were detected using the CCK-8 assay after treatment with different concentrations of EB. **D**-**G**, Cell colony-forming ability of MG63 and U2OS cells after treatment with EB (100 µM). **H**-**K**, Cell migration capacity of MG63 and U2OS cells following treatment with EB (100 µM). Scale bars, 200 μm. Data are reported as means±SD. *P<0.05, **P<0.01, ***P<0.001, ****P<0.0001. The data of CCK-8 experiments were analyzed by analysis of variance of factorial design. The data of colony-formation experiments were analyzed by Student's *t*-test.

Numerous drugs, such as paclitaxel, were derived from natural plant species and eventually incorporated into standard therapy regimens (11,12). Eriocalyxin B (EB), identified by Handong Sun's group in 1982, is a natural ent-kaurene diterpenoid compound extracted from the traditional Chinese herb *Isodon eriocalyx var*. *laxiflora*, which is predominantly distributed in Southwest China (13) (Figure 1A). Recent studies have reported that EB displays multifunctional activities, including modulating various biological processes, such as anti-inflammatory processes in Crohn's disease-like colitis by modulating the JAK2/STAT1 signaling and inhibiting adipogenesis via cell cycle arrest. Furthermore, EB suppresses malignant behaviors such as proliferation, migration, and invasion in several types of cancers, including breast cancer, prostate cancer, colorectal cancer, and ovarian cancer (14-22). Notwithstanding, most research focusing on the anti-tumorigenic activity of EB is limited to *in vitro* experiments, and the exploration of its mechanism has largely concentrated on apoptosis, cell cycle arrest, angiogenesis, and autophagy. In other words, studies on anti-tumorigenic effects of EB are scarce. Considering previous reports regarding the multifunctional activity of EB, exploring and validating its role in OS holds considerable implications.

Transcription elongation factor a3 (TCEA3) belongs to the TFIIS (TCEA) family and is one of the best-characterized transcription elongation factors (23). Its role in tumor pathology and development has attracted extensive attention, with earlier studies documenting that it can serve as a potent tumor suppressor owing to its ability to facilitate apoptosis in rhabdomyosarcoma, gastric cancer, and ovarian cancer via multiple signaling pathways (24-26). However, its role in OS remains largely unknown.

In the present study, a series of *in vitro* and *in vivo* experiments were performed to explore the effect and mechanism of EB in pathogenesis and immunotherapy in OS.

## Material and Methods

### Cell lines

Human normal osteoblast cell line (hFOB1.19), human OS cell lines (MG63, U2OS, Saos-2, and HOS), and murine OS cell line K7M2 were purchased from Cellcook (China), originating from American Type Culture Collection (ATCC, USA), which also supplied the STR identification report for all cell lines. All cell lines have undergone mycoplasma tests by MycAway™ Mycoplasma Real-time qPCR Detection Kit (Cat#40618ES25, YeSen, China) and were routinely cultured in Dulbecco's modified Eagle's medium (Cat#C11995500BT,DMEM; Gibco, USA) containing 10% fetal bovine serum (FBS,Cat#10091148,Gibco) at 37°C in a 5% CO_2_ humidified incubator. hFOB1.19 was cultured in DMEM/F12 with 10% FBS at 34°C in a 5% CO_2_ humidified incubator.

### Animal experiments

To construct subcutaneous tumor models, 1 × 10^6^ MG63 and U2OS cells were added to 100 µL PBS and subcutaneously injected into BALB/c-nude mice (female, 4-5 weeks old, 16-18 g). In addition, 0.5-1 × 10^6^ K7M2 cells were added to 100 µL PBS and then subcutaneously injected into wild-type BALB/c mice (female, 5-6 weeks old, 18-20 g). Tumor volumes were measured and calculated using the formula: 0.5 *A * B^2^ (A: length; B: width). Upon attaining a volume of 50 mm^3^, the engrafted tumors were treated according to the following protocol: PD1ab (BioXCell (USA) Cat#BE0146, 250 µg/per mouse, *ip* 1 × /2days), CTLA4ab (BioXCell, Cat#BE0146, 100 µg/per mouse, *ip* 1 × /2days), or IgG isotype control (BioXCell, BE0089, *ip* 1 × /2days) and Eriocalyxin B (MedChemExpress (USA), Cat#HY-N2303,10 mg/kg, *ip* 1 × /day). After being anesthetized by pentobarbital, the mice were euthanized upon attaining a tumor volume of roughly 1000 mm^3^ or evidence of ulceration was observed, which occurred approximately 14-16 days after cell injection. Lastly, the subcutaneous tumors were excised and fixed in formalin (10%) for 48 h.

### Immunohistochemistry

All tumor tissues were initially fixed in formalin (10%) and cut into sections. Each tumor section was deparaffinized and dehydrated by utilizing sodium citrate buffer and hydrogen peroxide (3%). Then, the sections were incubated with primary antibodies (overnight) and the appropriate secondary antibodies for 1 h. After washing with PBS three times, the sections were incubated with the ABC solution, followed by 3,3′-diaminobenzidine (DAB) staining. Lastly, the sections were dehydrated using xylene and mounted using neutral glue. The sections were photographed, and the number of positive cells was counted. The antibodies used in this experiment were as follows: CD8 (1:1000, Cat#ab209775, Abcam, USA),Ki67 (1:400, Cat#ZM-0166, ZSGB-BIO, China), and secondary antibody (1:2000, Cat#7074, Cell Signaling, USA).

### CCK8 assay

MG63 and U2OS were first seeded onto 96-well plates at a density of 200 cells/well. Next, various concentrations of EB (0,10, 100 µM) were added to the wells, and the cell culture was incubated with the CCK-8 reagent (Cat#C0037, Beyotime, China) for pre-defined durations (1, 2, 3, 4, 5, and 6 days). Cell survival was evaluated using a microplate reader (Bio-Rad Laboratories Inc., USA) by measuring the absorbance value at 450 nm.

### Colony formation assay

MG63 and U2OS cells were initially seeded onto 6-well plates containing 100 µM EB at a density of 500-800 cells/well and incubated for 10-14 days. On days 6-7, the medium was replaced by DMEM supplemented with 10% fetal bovine serum. On days 10-14, visible cell colonies were fixed in methyl alcohol and stained with Giemsa dye. Finally, the plates were dried and photographed, and the number of cell colonies was counted by ImageJ software (NIH, USA).

### Wound healing assay

MG63 and U2OS cells were initially seeded onto 6-well plates at a density of 10^4^ cells/well. Upon the formation of a cell monolayer, wounds in the cell layer were created using a 10-µL pipette point, and the cultured medium was replaced with a medium containing 100 µM EB. The straight lines were photographed at specified time points (0, 12, 24, and 36 h), and the migrated cells were independently counted by two individuals (L.Q.Z. and M.L.C.).

### Quantitative real-time PCR

MG63 and U2OS cells were seeded onto 6-well plates at a density of 10^4^ cells/well. On the next day, 0, 10, 50, and 100 µM EB was added to the wells. After 48 h, the cells were collected using TRIzol reagent (Invitrogen, USA). The PrimeScript RT reagent kit (TaKaRa, Japan) was utilized to reverse transcribe the mRNA of EB-treated cells. SYBR Premix ExTaq (TaKaRa) was employed to carry out the qPCR assay on the Applied Biosystems 7500 System. mRNA expression levels were quantified using the 2^−ΔΔCt^ method. The sequences of the TCEA3 mRNA primers were as follows: 5′-CCCCAAAACACCTAGCAGC-3′ (forward) and 5′-CTTCATGTCCGTGCTCTTGAG-3′ (reverse) (synthesized by RIBOBIO, China). The mRNA expression of GAPDH was chosen to be the internal parameter and the primers were as follows: 5′-TGTGGGCATCAATGGATTTGG-3′ (forward) and 5′-ACACCATGTATTCCGGGTCAAT3′ (reverse).

### Western blotting analysis

Total cell proteins were separated by SDS-polyacrylamide gel electrophoresis (PAGE) and electrotransferred onto a polyvinylidene difluoride (PVDF) membrane (Pall Corp. USA). The membranes were then blocked with 5% skimmed milk and incubated using primary antibodies against TUBULIN (1:1000 dilution, Cat#RM2003) and anti-TCEA3 (1:1000 dilution, Cat#MA5-24565), followed by incubation with the appropriate secondary antibodies. An enhanced chemiluminescence (Pierce, USA) was used to detect signals.

### Cell cycle analysis

For cell cycle analysis, ∼10^6^ cells were collected and fixed in 70% ethanol at 4°C overnight. Cells were washed with PBS and incubated with propidium iodide and RNase A solution (9:1; Cat#KGA512, KeyGEN BioTECH, China) following manufacturer instructions. Fluorescence at 488 nm wavelength was detected and analyzed using BD LSRFortessa X-20 cell analyzer and BD FACSDiva (Becton Davidson and Co., USA).

### Cell transfection

Briefly, MG63 and U2OS cells were seeded onto 6-well plates and cultured in an incubator overnight. Upon reaching 70-80% confluence, cells were transfected with the siRNA targeted TCEA3. The sequences were as follows: CCATCCAGCTA, CTACAGACAA, synthesized by RIBOBIO Institute, using Lipofectamine 2000 (Cat#11668019, ThermoFisher, USA). The efficiency of RNA interference and overexpression was determined via qPCR.

### Statistical analyses

The data were recorded and processed using EXCEL software (Microsoft, USA), whilst GraphPad Prism 9.02 software (USA) was employed for statistical analyses. Data are reported as means±SD. The Student's *t*-test was used for between-group comparisons. P<0.05 was considered statistically significant.

## Results

### EB inhibited the proliferative, colony-forming, and migratory abilities of human OS cells

In the current study, EB significantly suppressed the proliferative ability of both human OS cell lines, namely MG63 and U2OS, and showed no significant toxicity to the normal osteoblast cell line hFOB1.19 at different concentrations (0, 10, 100 µM) (Figure 1B and C, Supplementary Figure S1A). Likewise, the results of the colony formation assay indicated that EB impaired the colony-forming ability of both MG63 and U2OS human OS cells (Figure 1D-G). EB attenuated cell cycle progression in both MG63 and U2OS human OS cells (Supplementary Figure S1B and C). Furthermore, the result of the wound healing assay using the MG63 cell line demonstrated that EB significantly inhibited the migratory ability of human OS cells (Figure 1H and I). At the same time, the inhibitory effect of EB on the migratory ability of OS cells was also examined by using U2OS human OS cells (Figure 1J and K). As a result of these findings, it can be concluded that EB can inhibit the proliferative, colony-forming, and migratory abilities of OS cells *in vitro*.

### EB suppressed the proliferative ability of human OS cells *in vivo*

**Figure 2:**
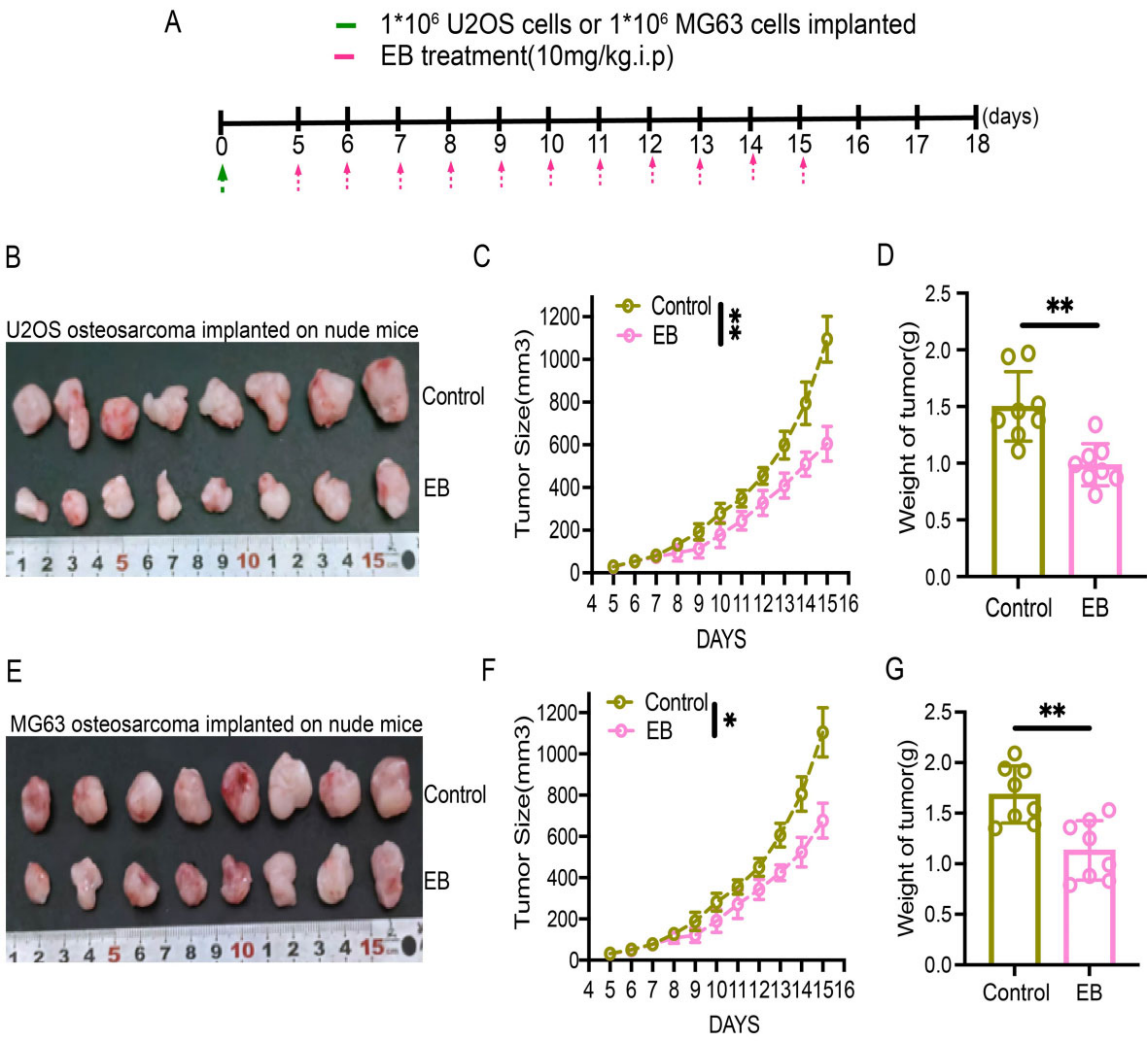
Eriocalyxin B (EB) inhibited human osteosarcoma (OS) growth *in vivo*. **A**, Schematic representation of the study protocol for the effect of EB treatment on U2OS or MG63 subcutaneous tumors in nude mice. **B**, Representative images of U2OS xenograft tumors. **C** and **D**, U2OS tumor growth curve and tumor weight in each group. **E**, Representative images of MG63 xenograft tumors. **F** and **G**, MG63 tumor growth curve and tumor weight in each group. Data are reported as means±SD. *P<0.05, **P<0.01. The data of tumor growth curve were analyzed by analysis of variance of factorial design. The data of tumor weight were analyzed by Student's *t*-test.

The results of the animal study demonstrated that in the MG63 OS model, mice in the EB-treated group possessed considerably smaller tumor mass and lower weight than those in the control group (Figure 2A-D). These results were consistent with those noted in the U2OS OS model (Figure 2E-G). Thus, our findings implied that EB could further suppress human OS growth *in vivo.*

### EB might suppress the progression of human OS cells by downregulation of TCEA3 expression.

**Figure 3:**
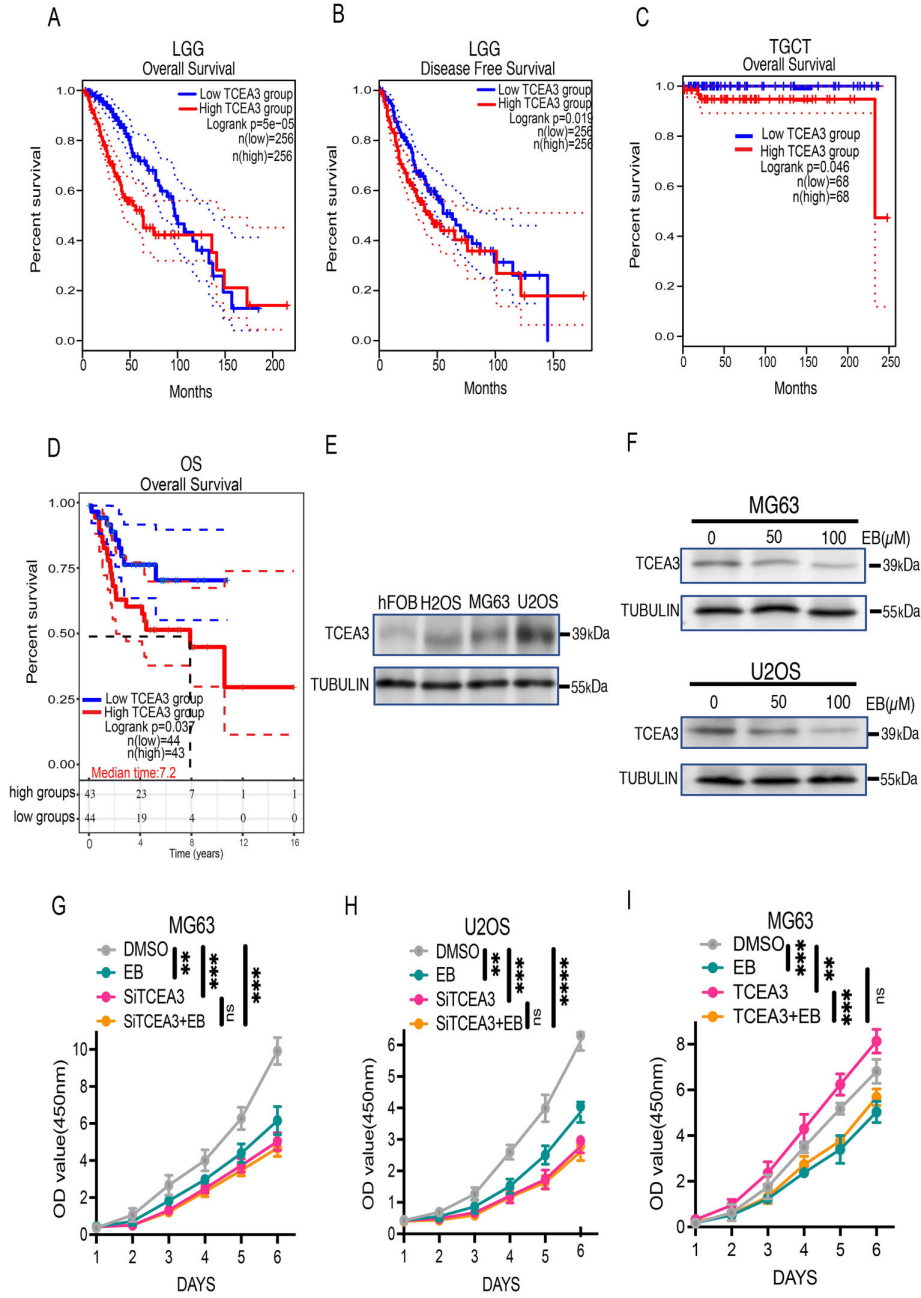
Eriocalyxin B (EB) might suppress osteosarcoma (OS) progression by downregulation of TCEA3 expression. **A** and **B**, Overall survival and disease-free survival analyses by TCEA3 expression in brain lower grade glioma (LGG) patients from TCGA datasets. **C**, Overall survival analysis by TCEA3 expression in testicular germ cell tumors (TGCT) patients from TCGA datasets. **D**, Overall survival analysis by TCEA3 expression in OS patients from TARGET datasets. **E**, Levels of TCEA3 expression in OS cell lines and a human normal osteoblast cell line (hFOB1.19). **F**, TCEA3 expression in OS cell lines MG63 and U2OS after EB treatment. **G** and **H**, Cell proliferation ability of MG63 (**G**) and U2OS cells (**H**) after downregulation of TCEA3 expression and EB (100 µM) treatment, n=3. **I**, Cell proliferation ability of MG63 after downregulation of TCEA3 expression and EB (100 µM) treatment, n=3. Data are reported as means±SD. **P<0.01, ***P<0.001, ****P<0.0001. The data of CCK-8 experiments were analyzed by analysis of variance of factorial design. The data of OS and DFS were analyzed by log-rank test.

TCGA database analysis showed that up-regulation of TCEA3 expression was negatively correlated with overall survival (OS) and disease-free survival (DFS) in brain lower-grade glioma (LGG) patients (A and B) and OS in testicular germ cell tumors (TGCT) (Figure 3C). More importantly, TARGET database analysis determined that up-regulation of TCEA3 expression was significantly negatively correlated with overall survival in OS patients (Figure 3D), indicating that the clinical significance of TCEA3 in the prognosis of OS patients cannot be overlooked. The result of western blot assay indicated that TCEA3 expression was significantly higher in human OS cells (MG63, U2OS, and HOS) compared with that in osteoblast cells (hFOB1.19) (Figure 3E). Furthermore, EB treatment significantly down-regulated the protein expression of TCEA3 both in MG63 and U2OS human OS cell lines (Figure 3F). The result of qPCR detection revealed that EB treatment down-regulated the expression of TCEA3 *in vivo* (Supplementary Figure S1A). According to the findings of the CCK8 assay, knocking down TCEA3 expression by targeted interference RNA (Supplementary Figure S1B and C) suggested that TCEA3 knockdown impaired the proliferative ability of MG63 cells compared with that of control cells. Contrastingly, this inhibitory effect was comparable between the si-TCEA3 group and EB+si-TCEA3 group (Figure 3G). In other words, EB did not suppress OS cell growth after knocking down the expression of TCEA3. Similar outcomes were observed in the human OS cell line U20S (Figure 3H). Furthermore, when compared with the control group, the TCEA3 overexpression group exerted a stimulatory effect on MG63 growth. However, compared with the TCEA3 overexpression group, the EB plus TCEA3 group showed a significant inhibiting effect (Supplementary Figure S1D, Figure 3I). In other words, EB neutralized the tumor stimulatory effect induced by TCEA3 overexpression. Indeed, the aforementioned results supported the hypothesis that EB might suppress OS progression by downregulating TCEA3 expression. Nonetheless, further studies are warranted to validate the aforesaid results.

### EB increased CD8^+^ T cell infiltration and sensitized ICBs therapy to OS *in vivo*

**Figure 4:**
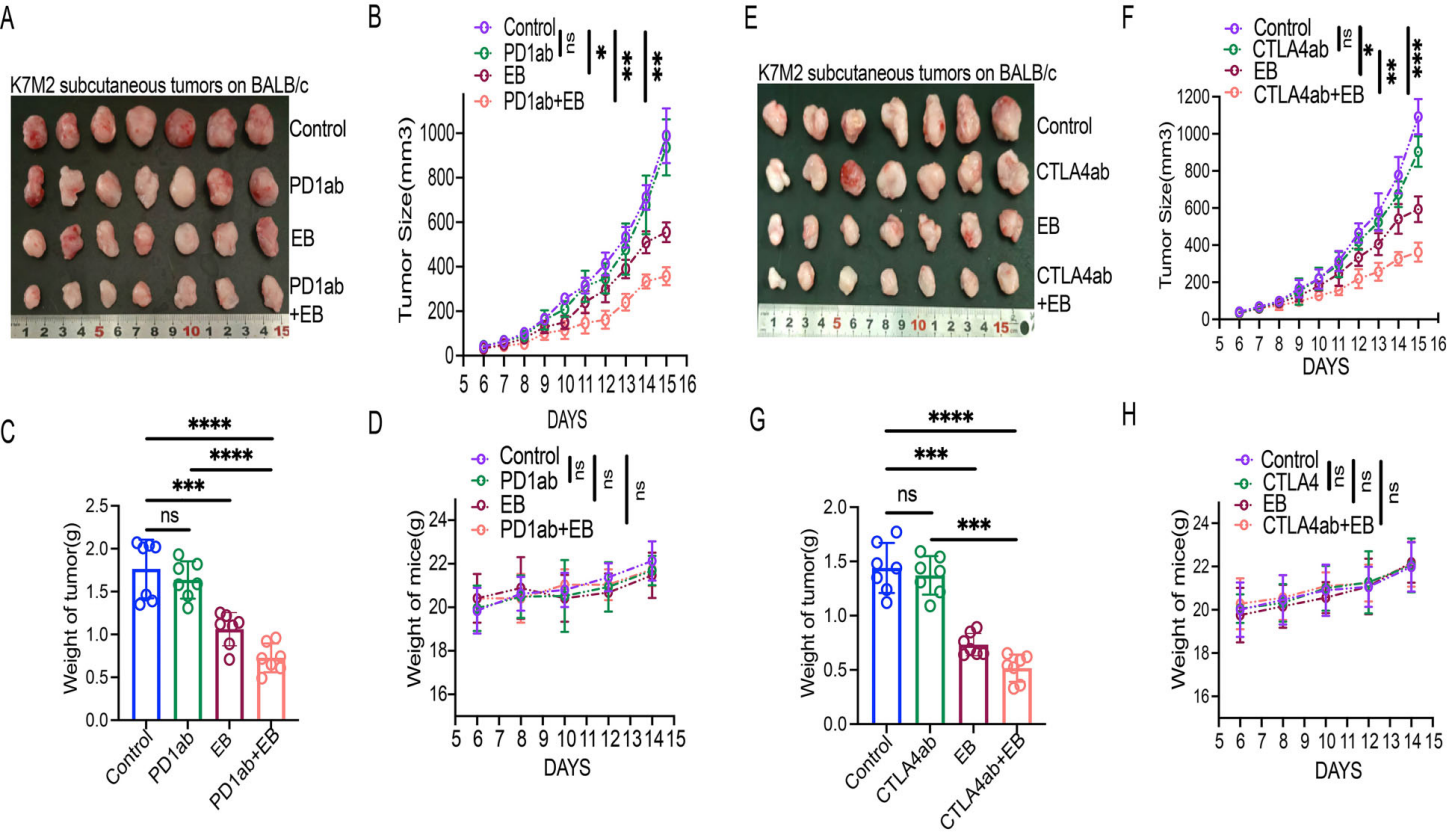
Eriocalyxin B (EB) enhanced the therapeutic efficacy of immune checkpoint blockades (ICBs) *in vivo*. **A**-**D**, K7M2 subcutaneous tumor models after treatment with PD1ab and EB. **A**, Representative images of tumors from each group, n=2; **B**, Tumor growth curve; **C**, Tumor weight; **D**, Weight of mice in each group. **E**-**G**, K7M2 subcutaneous tumor models after the administration of CTLA4ab and EB. **E**, Representative images of tumors in each group, n=2; **F**, Tumor growth curve; **G**, Tumor weight in each group; **H**, Weight of mice in each group. Data are reported as means±SD. *P<0.05, **P<0.01, ***P<0.001, ****P<0.0001, ns: not significant. The data of tumor growth curve were analyzed by analysis of variance of factorial design. The data of tumor weight were analyzed by Student's *t*-test.

EB is hypothesized to be directly involved in inhibiting the progression of various types of cancer (17,19,27,28). Conversely, its role in the tumor environment, especially in the immune environment, is underexplored. In this study, a subcutaneous K7M2 tumor model was constructed in immunocompetent mice, and the findings revealed that the inhibitory effect was similar between the control group and the PD1ab group, whereas significant differences were observed in the inhibitory effect of the EB group. Furthermore, compared with the PD1ab group, the therapeutic effect was higher in the EB+ PD1ab group (Figure 4A-C). Interestingly, EB and PD1ab treatment did not significantly alter the weight of mice (Figure 4D). There were no significant differences between tumor-bearing mice receiving CTLA4ab and those in the control group, whereas OS growth was limited in mice receiving EB (Figure 4E). In addition, the EB+ CTLA4ab group exhibited a slower tumor growth rate and lower tumor weight than the CTLA4ab group (Figure 4F-G). The weight of mice was comparable across the groups (Figure 4H).

**Figure 5:**
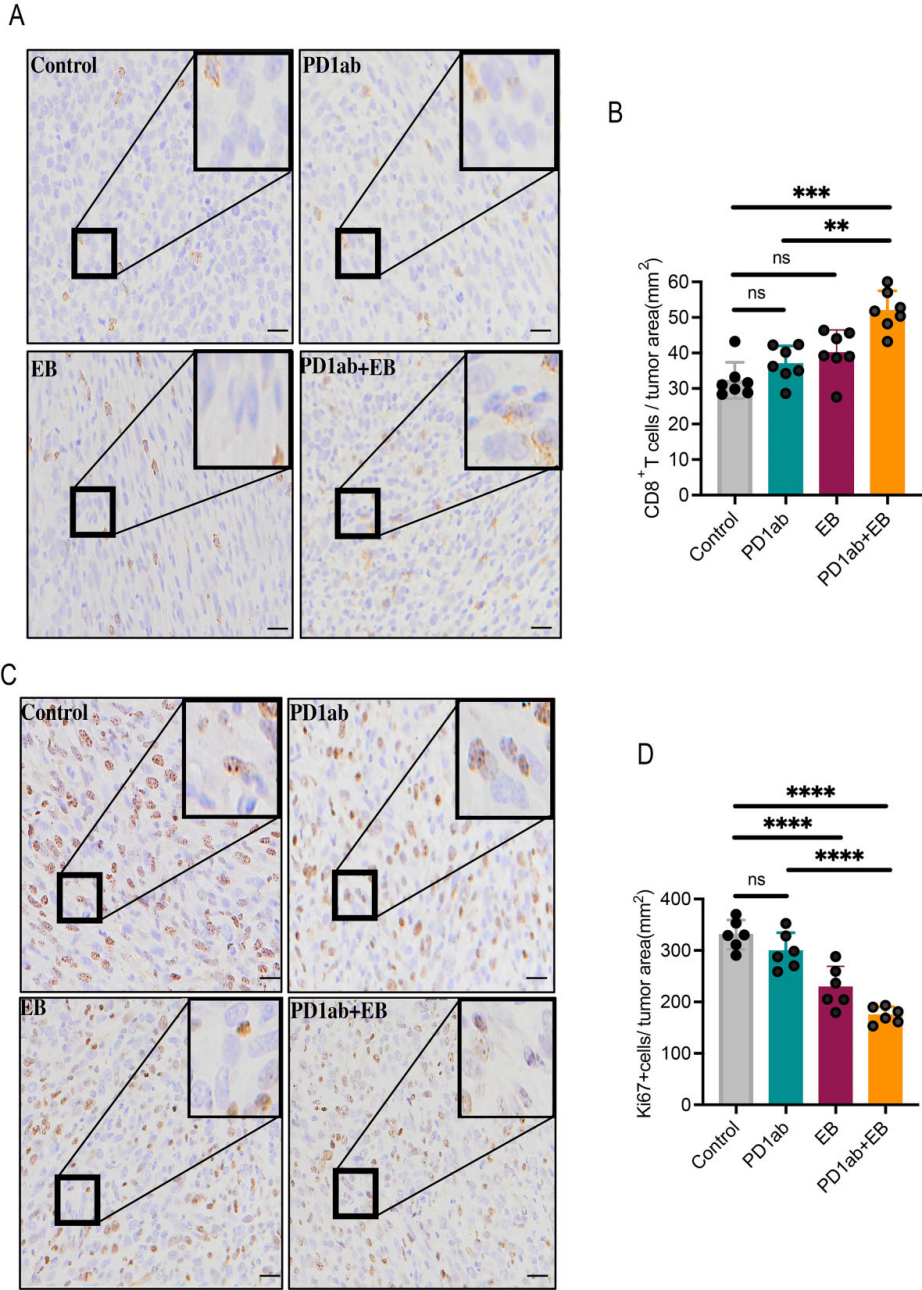
Immunohistochemical analysis in osteosarcoma (OS) tissue from K7 subcutaneous tumor models following PD1ab and Eriocalyxin B (EB) treatment. Representative images (**A**) and quantitative analysis (**B**) of CD8^+^T cells in each group, n=2 and representative images (**C**) and quantitative analysis (**D**) of Ki67^+^ cells in each group, n=2. Scale bars: 100 μm. Data are reported as means±SD. **P<0.01, ***P<0.001,****P<0.0001, ns: not significant (Student's *t*-test).

Immunohistochemistry analysis of K7M2 tumors demonstrated that the CD8^+^ T cell infiltration level was higher while the expression level of Ki67 was lower following EB treatment compared with the control group. As anticipated, CD8^+^ T cell infiltration levels were improved while Ki67 expression was disrupted in the tumor microenvironment of the EB+ PD1ab group compared with the PD1ab group (Figure 5A-D). The CD8^+^ T cell infiltration level was also higher in EB+CTLA4ab group compared with the CTLA4ab group (Supplementary Figure S1D and E).

## Discussion

Herein, EB inhibited the proliferative, colony-forming, and migratory abilities of human OS cells by downregulating TCEA3 expression both *in vitro* and *in vivo*. Furthermore, the combination of EB and the ICBs PD1ab and CTLA4ab significantly enhanced anti-tumorigenic activity by driving CD8^+^ T cell infiltration and inhibiting the expression of Ki67 in immunocompetent mice with OS. To the best of our knowledge, this is the first study to explore the activity and mechanism of action of EB in OS both *in vitro* and *in vivo*. In summary, these findings suggested that EB is a promising therapeutic or preventive agent for the treatment of OS.

Cancer research on EB largely focuses on its anti-tumorigenic action and the direct inhibition of cancer proliferation. For instance, a prior report described that EB suppressed triple-negative breast cancer metastasis through reducing aldehyde dehydrogenases 1 family member A1 (ALDH1A1) expression both *in vitro* and *in vivo* (16). Another study documented that EB covalently modified glutathione and selectively inhibited thioredoxin reductase, thereby inducing potent oxidative stress-mediated apoptosis in colorectal carcinoma RKO cells (18). Importantly, our findings corroborated the anti-tumorigenic activity of EB and indicated that its tumor-inhibitory effects may extend to various types of cancer.

TCEA3 is a vital transcription elongation factor belonging to TFIIS in vertebrates (29). Of note, it has been shown to modulate diverse biological processes, such as promoting muscle cell differentiation and inhibiting cardiomyocyte hypertrophy via multiple signal pathways (30,31). Nonetheless, its role in tumor pathogenesis and development remains controversial and largely elusive. Earlier studies claimed that TCEA3 could serve as a potent tumor suppressor, given that it contributed to apoptosis in rhabdomyosarcoma, gastric cancer, and ovarian cancer via multiple signaling pathways (24-26). Moreover, researchers recently discovered that TCEA3 acts as a tumor promoter in colorectal cancer. In this previous study, TCEA3 was found to attenuate pyroptosis and apoptosis of colorectal cancer cells induced by chemotherapeutic doxorubicin (32). In our study, the oncogenic role of TCEA3 was supported by the detection of TCEA3 expression in osteoblasts and OS cells. Furthermore, after knocking down TCEA3 expression, EB could not significantly suppress OS progression. Collectively, these results established that TCEA3 may play an oncogenetic role as well as mediating the effect of EB in OS.

**Figure 6:**
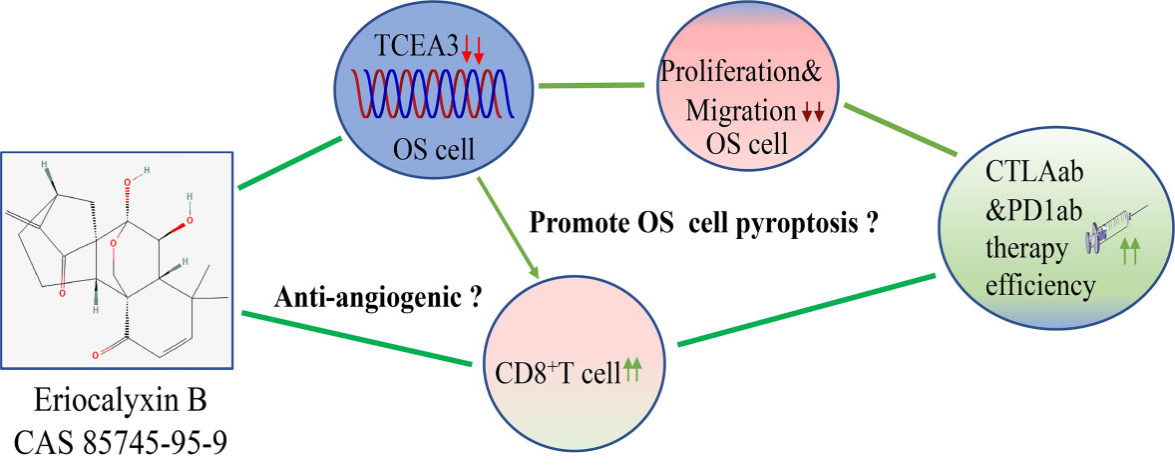
Schematic diagram illustrating that the natural product Eriocalyxin B (EB) exerted anti-tumor activity via downregulation of TCEA3 and sensitized immune checkpoint blockades therapy in osteosarcoma.

Owing to its outstanding efficacy and reduced side effects, immunotherapy has attracted significant attention and is currently considered a potent strategy for cancer therapy (33). Immunotherapies remain as a promising strategy for OS including ICBs, adoptive T-cell therapy, cytokines, and cancer vaccines. These strategies aim to restore immunity by reactivating the suppressed immune environment in OS patients and targeting immune cells (34). Among them, ICBs such as PD-1, PD-L1, and CTLA-4 inhibitors have been reported to exert optimal therapeutic effects in various malignant tumors with a lower frequency of adverse events than conventional therapy such as chemotherapy, especially for the treatment of melanoma, bladder cancer, lung cancer, and hematopoietic malignancies (35-38). However, the majority of OS patients do not benefit from ICB therapy owing to the severely restricted immune environment in OS (39). Herein, experiments involving the murine OS model demonstrated that EB increased CD8^+^ T cell infiltration levels and sensitized the effectiveness of ICBs, namely PD1ab and CTLA4ab therapies, *in vivo*. These effects may be associated with EB increasing the permeability of vital immune cells such as CD8^+^ T cells owing to its previously reported anti-angiogenic activity. Considering a recent report, which indicated that knockdown of TCEA-3 enhanced pyroptosis in colorectal cells (35), and that pyroptosis showed a close relationship with the infiltration and function of CD8^+^ T cell (40), we may also hypothesize that EB sensitizes ICB therapy through TCEA3-medicated pyroptosis (Figure 6). However, detailed studies should be conducted in the future.

## Supplementary Material

Click to view [pdf].
