# Supplementary material for: Natural product Eriocalyxin B exerts anti-tumor effects by downregulating TCEA3 expression and sensitizes immune checkpoint blockade therapy in osteosarcoma
Source: Braz J Med Biol Res. 2025 Jan 31;58:e14112. doi: 10.1590/1414-431X2024e14112 (PMC11793143; doi:10.1590/1414-431X2024e14112)

Figure S1. A, Cell proliferation ability of hFOB1.19 using the CCK-8 assay after treatment with different concentrations of Eriocalyxin B (EB). **B**, Cell cycle distribution analysis of MG63 after EB (100 μM) treatment for 48 h. Fraction of cells in G1/G2/S phase was demonstrated. **C**, Cell cycle distribution analysis of U20S after EB (100 μM) treatment for 48 h. Fraction of cells in G1/G2/S phase was indicated. Representative images (**D**) and quantitative analysis (**E**) of CD8+T cells in each group, n=2. Scale bars: 100 μm. Data are reported as means±SD. *P<0.05, ***P<0.001, ns: not significant. Number of CD8+ T cells and Ki67 + cells were statistically analyzed by Student's *t*-test.

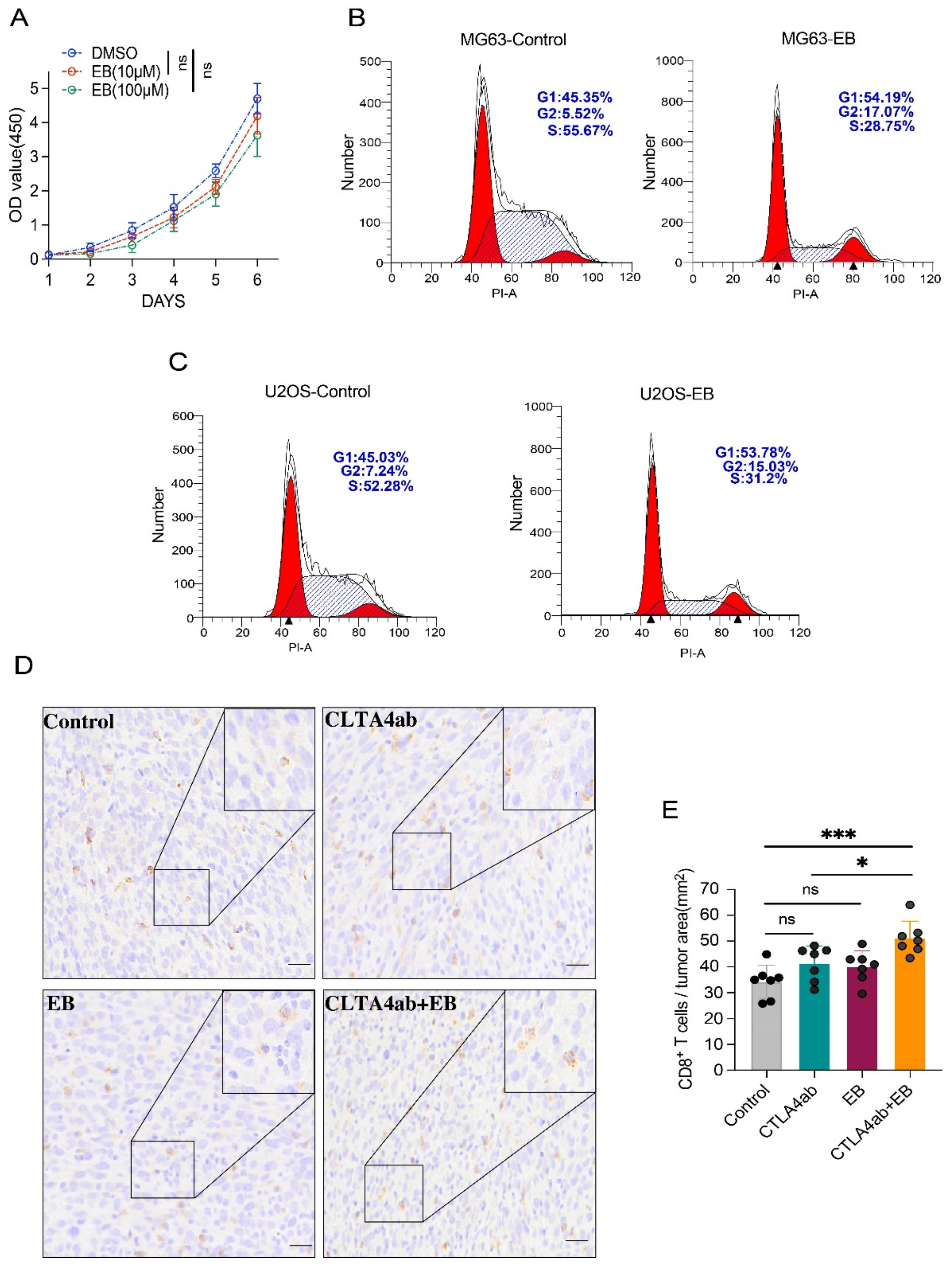

Supplement: Supplementary file 1 [file 1414-431X-bjmbr-58-e14112-suppl.pdf]
